# Managing a wooden foreign body in the neck

**DOI:** 10.4103/0974-2700.55340

**Published:** 2009

**Authors:** Rakesh Kumar Singh, Sangita Bhandary, Prahlad Karki

**Affiliations:** Department of Otolaryngology Head and Neck Surgery, B P Koirala Institute of Health Sciences, Dharan, Nepal; 1 Department of Internal Medicine, B P Koirala Institute of Health Sciences, Dharan, Nepal

**Keywords:** Foreign body, management, outcome, wooden

## Abstract

An interesting case is presented of a wooden foreign body in the neck entering through the right lower vestibule of the mouth. The foreign body traveled subcutaneously in the neck and got stuck in the clavicle, without damaging any vital structures. In this case, the authors highlight the mode of entry of the foreign body, the peculiarity of the wooden foreign body, the management protocol and the outcomes of the penetrating neck injury by the wooden stick.

## INTRODUCTION

Foreign bodies may be ingested, inserted into the body cavity or deposited inside by a traumatic or iatrogenic injury. The penetrating foreign body in the neck has a special apprehension because of the constellations of vital structures in the neck.[1] The wooden foreign body creates more diagnostic difficulties. It may initially disappear in the computerized tomography (CT) scan. The management could be difficult due to its easy fragility and infectious nature.[2] The early wound exploration and proper wound debridement with proper antimicrobial coverage are the prime factors in curtailing morbidity and mortality. Irrespective of these problems, the overall mortality is relatively low, ranging from 0 to 11%.[3]

## CASE REPORT

**Figure 1:**
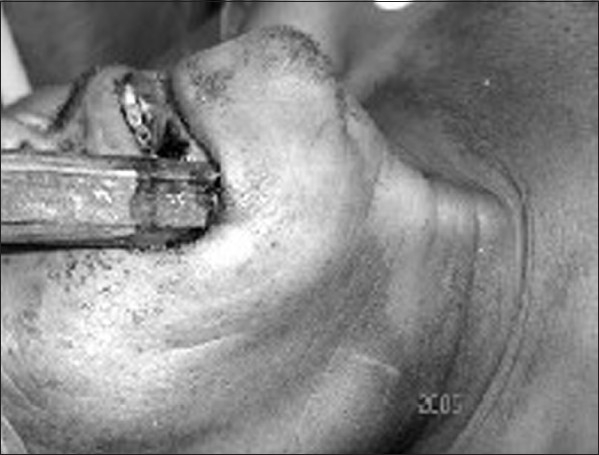
Penetrating wooden stick in the neck protruding from the mouth

A 51-year-old drunken male lost his balance and went fell down on the ground with a wooden stick in his hand. The pointed end of the wood impaled through the right lower vestibule of his mouth, traveled subcutaneously in the neck and was stuck in the clavicle. There was no excessive bleeding, vomiting or loss of consciousness and respiratory distress. The relatives of the patient did not make any attempt to remove the protruding wooden piece. Within 3 h of the primary incidence, the patient was transported to the emergency department of the B P Koirala Institute of Health Sciences, Dharan, Nepal. The patient did not have any history of dyspnea, dysphagia, oronasopharyngeal bleeding and neurological deficit. Mild trickling of blood from the entry site was noticed in the hospital. The arterial pressure was 130/86mmHg and the pulse rate was 80/min. The bamboo stick was seen protruding through his mouth [Figure 1]. The entry point was 3cm longer in transverse diameter in between the mandible and the lower lip toward the right side. A hard 13cm long and 4cm wide longitudinal swelling extending from the under lip to the clavicle was palpated [Figure 1]. It was found that the distal end of the wooden stick moved with movement of the proximal end. No other swelling was revealed in other part on the neck. A further examination of the neck revealed the absence of subcutaneous emphysema, bruit, thrill, stridor and respiratory distress. The pulsation of the carotid and superficial temporal artery was intact bilaterally and was also absolutely symmetrical in radial and brachial pulse. The expansion of the chest and the bilateral air entry in the chest were normal. The remaining ENT and systemic examinations were within the normal limit.

**Figure 2:**
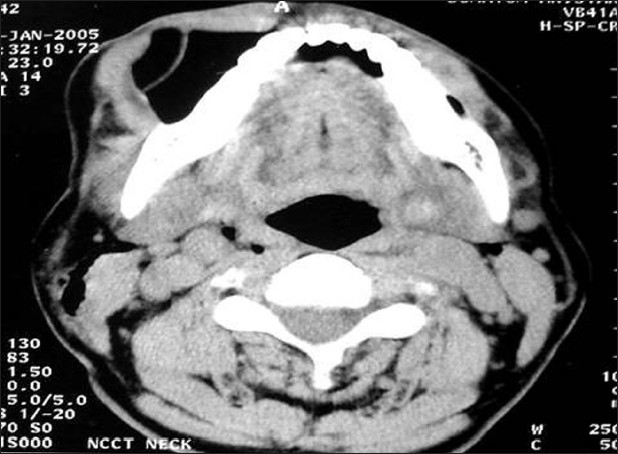
Axial non-contrast computerized tomography scan showing a hypodense object mimicking an air bubble lateral to the mandible on the right side

Immediately, non-contrast CT followed by contrast-enhanced CT (CECT) scan of the head and neck region were performed, which revealed a low-density area with a density (Hounsfield Unit, -1003) equivalent to air under the soft tissue and lateral to the mandible on the right side [Figure 2]. The mandible, hyoid, clavicle and cartilaginous framework of the larynx and trachea were normal in texture. No fracture in related bones and cartilages was revealed by the CT scan. There was no evidence of vascular injury and hematoma formation. The contrast CT angiography revealed normal integrity of the major vessels. The CT scan of the thorax was absolutely within the normal limit.

**Figure 3:**
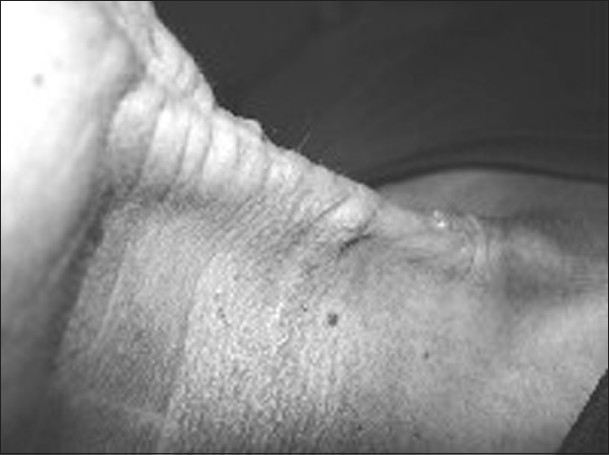
A thick fibrous band along the line of incision

In the operation theater and under general anesthesia, the wound was explored by a vertical incision along the long axis of the foreign body. The bamboo stick was removed completely, a corrugated drain was placed and the wound was closed in layer after securing hemostasis. The foreign body did not damage any major structures, including the major blood vessels. Intravenous antibiotics, anti analgesic and prophylaxis of tetanus were given in appropriate doses. The drain was removed on the third postoperative period and the patient was discharged on the seventh day. Follow-up after the fourth week revealed a thick fibrous band and wound contracture along the line of incision [Figure 3]. The wound was re-explored and the fibrous tissue and scar were completely removed. The subsequent follow-up revealed complete healing of the wound.

## DISCUSSION

Since time immemorial, foreign bodies would have been a lowbrow amusement to the clinician as well as to the population. Sometimes it may turn out uneventfully; otherwise it endangers the life of the patient depending on the type, size and location of the foreign body. The penetrating injury of the neck with a bamboo may pose a diagnostic and therapeutic dilemma. Thorough knowledge of the anatomy of the neck, physical assessment and current recommendations for diagnostic and therapeutic interventions are necessary for appropriate management.

### Applied anatomy of the neck

The neck is a complex region from an anatomical point of view. It contains a constellation of a number of vital structures such as vascular, aerodigestive and neural, being protected by strong musculature, tough fascia and the related bones. The aggregation of these vital structures located in a small volume make them more vulnerable for damage in any penetrating injury of the neck crossing the platysma and can have serious and life-threatening consequences. However, in some places, the vital structures are protected with a tough, bony shield-like mandible and clavicle whereas in other places these are more superficial and can open up. Diversity of this arrangement compels the clinician to adopt a different management protocol for a different location of the neck. Keeping these facts in mind, the neck is anatomically divided into three zones, running from the inferior to the superior. Zone I is the horizontal area between the clavicle/suprasternal notch and the cricoid cartilage. The proximal common carotid, vertebral and subclavian arteries and the trachea, esophagus, thoracic duct and thymus are located in Zone I. Zone II is the area between the cricoid cartilage and the angle of the mandible. It contains the internal and external carotid arteries, jugular veins, pharynx, larynx, esophagus, recurrent laryngeal nerve, spinal cord, trachea, thyroid and parathyroids. Zone III is the area that lies between the angle of the mandible and the base of the skull. It has the distal extracranial carotid and the vertebral arteries and the uppermost segments of the jugular veins.[3–6] In the present case, the injuries were confined to the lower face, Zone I and Zone II, without damaging major vessels as well as the aerodigestive tract.

### Pathophysiology and natural history in penetrating neck injuries

Penetrating injuries to the neck are associated with high morbidity and mortality rates owing to the multiple vital structures present within this anatomic region. Foreign body in the neck would damage vital structures such as major blood vessels, the aerodigestive tract and the thyroid gland.[1,7] However, the course of stab wounds is more limited than that of gunshot wounds and there is still a clear potential for major injury. The depth and nature of the injury are highly determined by the kinetic energy delivered by object itself or by the subject.[1,2] The alcoholic status of the person is the prime factor now a days to inflicting the foreign substances inside the body. Most of the time, the person gets it through the mouth, which lodges the foreign body inside the aerodigestive tract. In some instances, the person loses his balance and gets the foreign body inside the body.[1,3]

After reviewing 130 subjects of penetrating neck injuries at the Canadian Trauma Center, Nason *et al.*[3] reported a single such case inflicted by a tree branch. In most of the instances, metal, broken glass and projectile was the common object causing penetrating foreign body in the neck.

Although a number of cases have reported on the penetration of the orbit or the head by a wood piece, it is skeptical to lodge a wooden in the neck, piercing through the vestibule of the mouth, as seen in this case. The blunt end of the wood piece, the resilience of the subcutaneous tissue and the resistance of the skin, deeper bones and muscles probably provide a way for the foreign body to travel in such a way. The axis of weight transmission and the direction of kinetic force during impact are the prime factors in determining the manner of foreign body transmission and, ultimately, the amount of tissue damage.[2,3,8]

### Features of wooden foreign body penetrating the neck

The wooden foreign bodies are dirty and infectious because the porous organic material provides good culture conditions for gram positive and gram negative bacteria, which may cause abscess formation. Apparently, even clean wood may cause a foreign body reaction. If the foreign material gets a chance through or communicates with the digestive tract, the wound contamination is sky scraping in particular. Any infection in the neck is grave for the patient.[2,8]

Penetrating trauma due to wooden stick in the proximity to the course of major arteries may result in vascular injury. The vascular injuries are easily detected when hard signs, such as absent pulses, arterial bleeding, expanding hematomas, vascular thrills, bruit or frank ischemic changes, are present. In the absence of these hard signs, the course of the wooden stick within 1cm of the course of the named vessels and the soft signs, such as stable hematoma, reduced capillary refill, pulse and nerve deficits, are predictive of vascular injury.[9,10] In the present case, the hard signs as well as the soft signs were not observed during a detailed examination. The track of the wooden stick was more than 1cm away from the major vessels. Thus, we selected to perform both plain and contrast CT scan rather than to go in for an angiography or Doppler sonography.

### Using radiology in penetrating neck injuries

Although a significant number of studies recommended the CT scan as the investigation of choice for the stable case of penetrating neck injury, other investigations only enlighten the clinician about the few facts of injury. The CT scan, however, provides extra information about the integrality of the aerodigastric tract, the neurovascular structures and the vertebral integrality as well as the course, tract and position of the foreign body in the neck.[11–13] However, where the wooden foreign body is concerned, it provided extra challenges in evaluation of the depth and amount of tissue injuries. It may only be recognizable on roentgenograms in 15% of the subjects. The wooden foreign body, present as a hypodense area on CT image, mimicked air bubbles, as revealed in the present case. The bamboo piece is being organized as many knots surrounded by a very thin cellulose bark and air. Several months later, it appeared with a much higher density on CT because the wood may have absorbed water from the surrounding tissue.[14,15]

Selective arterial angiography is an important tool in diagnosing vascular injury in the case of penetrating neck injury. However, the use of angiography in patients in a stable condition with penetrating neck trauma has also been questioned because of the reported high (range, 70-90%) number of negative examination results. In fact, the percentage (about 1%) of missed vascular injuries using physical examination screening criteria is similar to the false-negative rate for angiography. Data from Ferguson and colleagues suggest that, in the absence of hard vascular signs with a penetrating neck injury, angiography is not necessary.[5,6,11] Keeping these facts in mind and considering the condition of our patient, we selected CT scan in our case.

In the absence of angiography, contrast-enhanced spiral CT scan, color Doppler ultrasonography and magnetic resonance angiography are the other radiological investigations that can provide important information about the vital structure injury. Each investigation has its own advantages and disadvantages.[7,12] The selection of a particular type of investigation depends on the financial condition of the patient, the available investigation modality in the tertiary health care center and the belief of the clinician on the different scientific studies on these modalities.

### Clinical management

**Figure 4:**
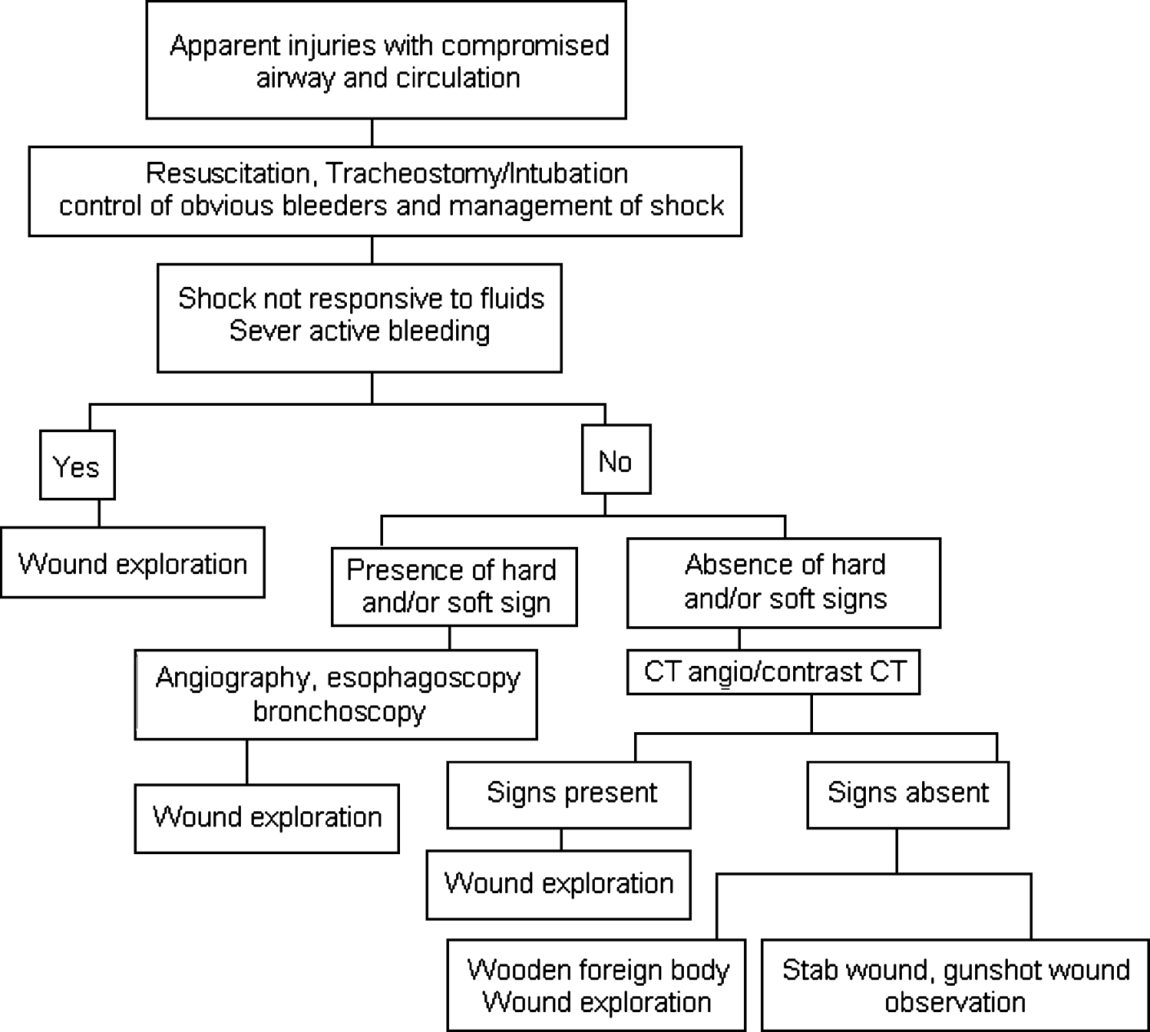
Algorithm for the evaluation and management of penetrating neck wounds

A remarkable number of changes have occurred in the treatment paradigm as new technologies have developed and as surgeons have explored the outcomes from different treatment protocols. Therapy has evolved from no treatment to non-operative management, to routine exploration, to selective exploration and adjunctive invasive or non-invasive assessment [Figure 4].[1,2,7,16,17] The patient may present in shock due to excessive bleeding or may present with respiratory distress. In such a case, the airway should be maintained by performing tracheostomy and shock should be managed with priority. All veins and external carotid artery injuries in the neck can be safely ligated to control hemorrhage. Internal jugular vein repair is mandated. Laryngeal and tracheal mucosal lacerations from the penetrating injury should be repaired early (within 24 h). Significant laryngotracheal-displaced cartilage fractures need surgical approximation. A soft laryngeal stent for the larynx and a T-tube stent for the trachea may be needed for badly macerated mucosa. When an esophageal injury is found early, management involves a two-layer closure with wound irrigation, debridgement and adequate drainage. After repair of the mucosal perforation, a muscle flap may be interposed over the esophageal suture line for further protection. Early radical debridgement and removal of the wood fragments are mandatory to prevent potentially fatal infectious complications. Postoperative intensive broad-spectrum antibiotic treatment should be administered to prevent late infectious events. Delay in surgical intervention and improper debridgement may lead to tissue necrosis, fibrosis and wound contracture.[1,3,7] In our case, we selected the option of early exploration of the neck as there was no evidence of major structural injury, as seen on CT scan of the neck and thorax. The vitals of the patient were within normal limits. There was damage to only a few superficially placed anterior jugular veins, which were ligated. Knowing the fact that the wooden stick contains multiple infectious organisms that can cause serious sepsis in the neck, including neck abscess and mediastenitis, the decision of early exploration and debridgement was made. Intravenous antibiotics, anti analgesic and prophylaxis of tetanus were given in appropriate doses.

### Prognosis

The current mortality rate for penetrating neck injury is 3-6%. The usual complications of penetrating neck injury are vascular trauma in 25%, with mortality rates approaching 50%. Tracheobronchial injuries may be observed in less than 10% to as high as 20% and a mortality rate of as high as 20% is also reported. The injured cervical esophagus may result in devastating complications such as leakage of saliva, bacteria, refluxed acid, pepsin and even bile. A 11-17% increase in the overall mortality rate has been observed after a delay of 12 h in the diagnosis of esophageal injuries.[3,4,16,17]

The present case is interesting because of the mode of entry of the foreign body, the way it travels under the subcutaneous tissue of the neck and the complication produced after management. The portal of entry of the bamboo piece was the right lower vestibule of the mouth that crossed over without damaging the alveolus, mandible, clavicle, visceral structure of the neck and major vessels, as detected by a CT scan of the neck and thorax. It traveled subcutaneously and was stuck in the clavicle. The main reason of thick fibrous tissue formation would be the longitudinal incision along the long axis of the foreign body and improper wound debridgement. To prevent such a complication, the wound should be explored by horizontal incision in the skin crease with proper wound debridgement.

## CONCLUSION

Penetrating foreign body in the neck is an uncommon but potentially life-threatening and crisis condition. Diversities in the management protocol with a changing technique compel the clinician to perform a close evaluation of the patient. Each maneuver should be directed to minimize mortality and morbidity by means of timely intervention. In cases of wooden foreign bodies, early exploration and removal reduces the chances of wound infection, resulting in a favorable outcome.

## References

[1] Hunter TB, Taljanovic MS (2003). Foreign Bodies. Radiographics.

[2] Singh RK, Bhandary S, Sinha BK, Karki P (2004). Penetrating injury of parotid gland and external auditory canal: A unique combination. J Laryngol Otol.

[3] Nason RW, Assuras GN, Gray PR, Lipschitz J, Burns CM (2001). Penetrating neck injuries: Analysis of experiences from a Canadian trauma centre. Canadian J Surg.

[4] Kendall JL, Anglin D, Demetriades D (1998). Penetrating neck trauma. Emerg Med Clin North Am.

[5] Ferguson E, Dennis JW, Vu JH, Frykberg ER (2005). Redefining the role of arterial imaging in the management of penetrating zone 3 neck injuries. Vascular.

[6] Beister P, Weigett JA, Flynn E, Easley S (1994). Physical examination and arteriography in patients with penetrating zone II neck wounds. Arch Surg.

[7] Múnera F, Soto JA, Palacio D, Velez SM, Medina E (2000). Diagnosis of arterial injuries caused by penetrating trauma to the neck: Comparison of helical CT angiography and conventional angiography. Radiology.

[8] Matsuyama T, Okuchi K, Nogami K, Hata M, Murao Y (2001). Transorbital penetrating injury by a Chopstick-case report. Neurol Med Chir.

[9] Weaver FA, Yellin AE, Bauer M, Oberg J, Ghalamber N, Emmanuel RP (1900). Is arterial proximity a valid indication for arteriography in penetrating extremity trauma? A prospective analysis. Arch Surg.

[10] Johansen K, Lynch K, Paun M, Copass M (1991). Non-invasive vascular tests reliably exclude occult arterial trauma in injured extremities. J Trauma.

[11] Gracias VH, Reilly PM, Philpott J, Klein WP, Lee SY, Singer M (2001). Computed tomography in the evaluation of penetrating neck trauma. Arch Surg.

[12] Hanpeter DE, Demetriades D, Asensio JA, Berne TV, Velmahos G, Murray J (2000). Helical computed tomographic scan in the evaluation of mediastinal gunshot wounds. J Trauma.

[13] Atteberry LR, Dennis JW, Menawat SS, Frykberg ER (1994). Physical examination alone is safe and accurate for evaluation of vascular injuries in penetrating zone II neck trauma. J Am Coll Surg.

[14] Imokawa H, Tazawa T, Sugiura N, Oyake D, Yosino K (2003). Penetrating neck injuries involving wooden foreign bodies: The role of MRI and the misinterpretation of CT images. Auris Nasus Larynx.

[15] Krimmel M, Cornelius CP, Stojadinovic S, Hoffmann J, Reinert S (2001). Wooden foreign bodies in facial injury: A radiological pitfall. Int J Oral Maxillofac Surg.

[16] Sekharan J, Dennis JW, Velder HC, Miranda F, Frykberg ER (2000). Continued experiences with physical examination ‘alone for evaluation and management o penetrating zone II neck injuries: Results of 145 cases. J Vas Surg.

[17] McConnell DB, Trunkey DD (1994). Management of penetrating trauma to the neck. Adv Surg.

